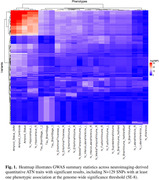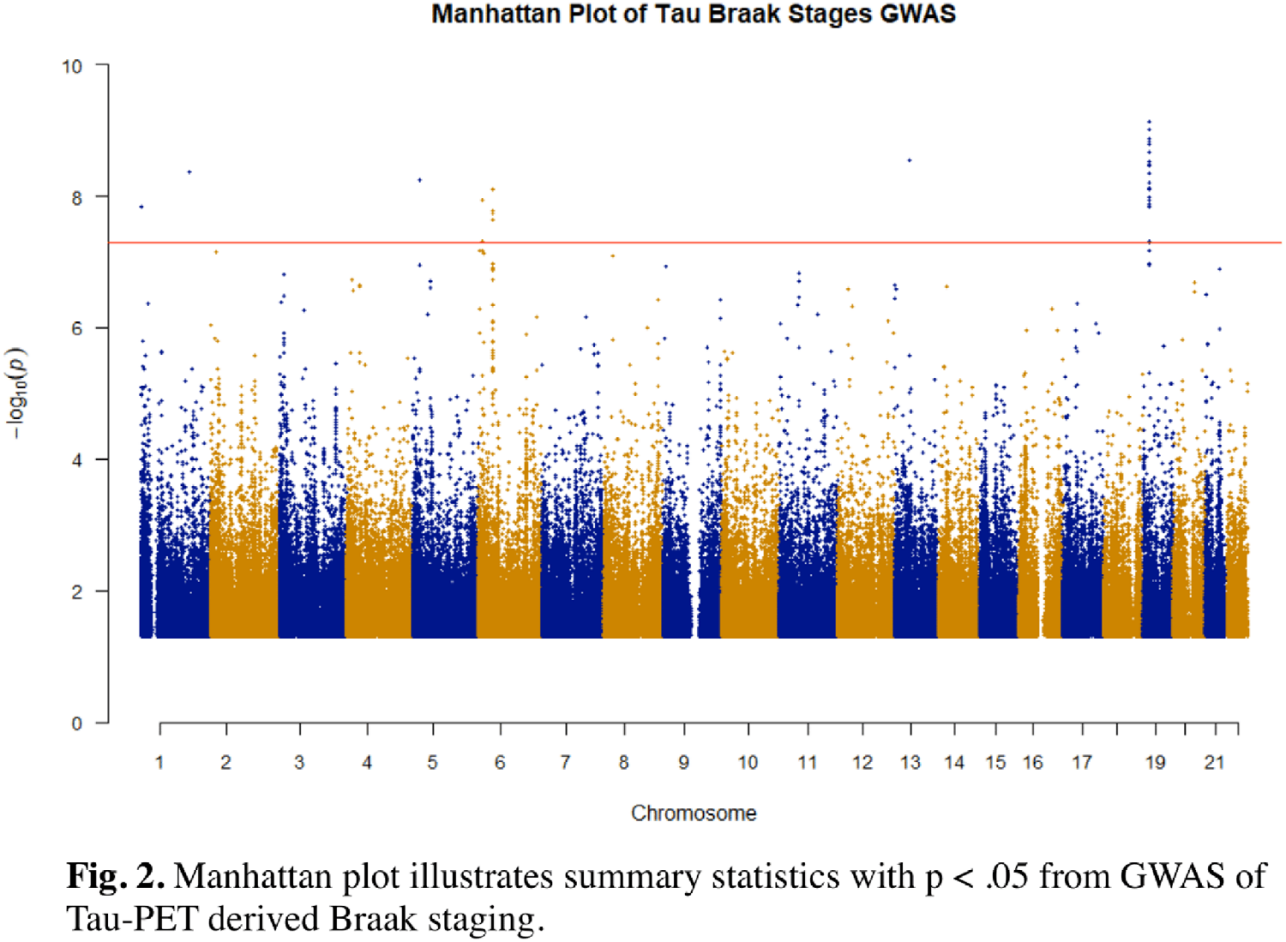# Genetic associations with amyloid, tau, and neurodegeneration: an ADSP neuroimaging study

**DOI:** 10.1002/alz.094870

**Published:** 2025-01-09

**Authors:** Elizabeth Mamourian, Jingxuan Bao, Yuhan Cui, Heng Huang, Andrew J. Saykin, Paul M. Thompson, Jason H. Moore, Marylyn D. Ritchie, Dokyoon Kim, Christos Davatzikos, Li Shen

**Affiliations:** ^1^ University of Pennsylvania, Philadelphia, PA USA; ^2^ University of Maryland, College Park, MD USA; ^3^ Indiana University, Indianapolis, IN USA; ^4^ University of Southern California, Los Angeles, CA USA; ^5^ Cedars‐Sinai Medical Center, West Hollywood, CA USA

## Abstract

**Background:**

The amyloid‐tau‐neurodegeneration (ATN) framework provides a valuable model for comprehending the pathophysiology and progression of Alzheimer’s disease (AD). However the relationship between and genetic interaction with these three characteristics are complex and not fully understood. Here, we use neuroimaging‐derived quantitative traits to evaluate the genetic risk for amyloid accumulation, tau pathology, and neurodegeneration.

**Method:**

The Alzheimer’s Disease Sequencing Project (ADSP) collected and harmonized whole genome sequencing (WGS) and quantitative phenotype data. This study examines harmonized neuroimaging traits, including 3 Amyloid‐PET features in N = 1,217 participants (Age = 72.6±8.1 years, Sex = 48.9% female), 6 Tau‐PET features in N = 470 participants (Age = 74.0±8.6 years, Sex = 53.8% female), and 20 selected T1 MRI derived volumetric measures of neurodegeneration in N = 2,644 participants (Age = 72.6±8.3 years, Sex = 52.0% female). We conducted genome wide association studies (GWAS) using Plink on the QC'ed WGS data of over 7.1 million variants for each trait, correcting for the top 10 principal components, age, and sex; for volumetric measures, we also control for intracranial volume.

**Result:**

We found associations at the genome‐wide significance threshold for all three characteristics, including N = 56 SNPs associated with Amyloid phenotypes, N = 53 SNPs associated with Tau phenotypes, and N = 77 SNPs associated with Neurodegeneration phenotypes (Fig. 1). The strongest associations to Amyloid phenotypes (amyloid positivity, centiloid values) overlap with Neurodegeneration phenotypes (hippocampal volume) in chromosome 19, with the lead SNPs located in *APOE*. We found an additional region on chromosome 6 significantly associated with Tau phenotypes (Braak staging), but not Amyloid or Neurodegenerative phenotypes (Fig. 2).

**Conclusion:**

These results provide additional evidence supporting the genetic contributions to risk of ATN pathology, confirming that variants in *APOE* and the surrounding region of chromosome 19 are strongly associated to amyloid positivity and hippocampal atrophy. These findings also support the genetic basis of a tau‐specific AD pathway. These results yield new insights into the genetic risk for amyloid accumulation, tau pathology, and neurodegeneration using the harmonized ADSP quantitative traits.